# Patterns of Genome-Wide Variation, Population Differentiation and SNP Discovery of the Red Banded Stink Bug (*Piezodorus guildinii*)

**DOI:** 10.1038/s41598-019-50999-z

**Published:** 2019-10-09

**Authors:** Maria I. Zucchi, Erick M. G. Cordeiro, Clint Allen, Mariana Novello, João Paulo Gomes Viana, Patrick J. Brown, Shilpa Manjunatha, Celso Omoto, José Baldin Pinheiro, Steven J. Clough

**Affiliations:** 1 Institute of Biology, University of Campinas, Campinas, SP Brazil; 2 Agência Paulista de Tecnologia dos Agronegócios, Pólo Regional Centro-Sul, Piracicaba, SP Brazil; 3 Department of Entomology and Acarology, University of Sao Paulo, Luiz de Queiroz College of Agriculture (USP/ESALQ), Piracicaba, SP Brazil; 4 US Department of Agriculture, Agricultural Research Service, Southern Insect Management Research Unit, Stoneville, MS USA; 5 Department of Crop Sciences, University of Illinois at Urbana-Champaign, Urbana, IL USA; 6 Department of Genetics, University of Sao Paulo, Luiz de Queiroz College of Agriculture (USP/ESALQ), Piracicaba, SP Brazil; 7 US Department of Agriculture - Agricultural Research Service, Urbana, IL USA

**Keywords:** Agricultural genetics, Structural variation

## Abstract

Unravelling the details of range expansion and ecological dominance shifts of insect pests has been challenging due to the lack of basic knowledge about population structure, gene flow, and most importantly, how natural selection is affecting the adaptive process. *Piezodous guildinii* is an emerging pest of soybean in the southern region of the United States, and increasingly important in Brazil in recent years. However, the reasons *P. guildinii* is gradually becoming more of a problem are questions still mostly unanswered. Here, we have genotyped *P. guildinii* samples and discovered 1,337 loci containing 4,083 variant sites SNPs that were used to estimate genetic structure and to identify gene candidates under natural selection. Our results revealed the existence of a significant genetic structure separating populations according to their broad geographic origin, i.e., U.S. and Brazil, supported by AMOVA (F_GT_ = 0.26), STRUCTURE, PCA, and F_ST_ analyses. High levels of gene flow or coancestry within groups (i.e., within countries) can be inferred from the data, and no spatial pattern was apparent at the finer scale in Brazil. Samples from different seasons show more heterogeneous compositions suggesting mixed ancestry and a more complex dynamic. Lastly, we were able to detect and successfully annotated 123 GBS loci (10.5%) under positive selection. The gene ontology (GO) analysis implicated candidate genes under selection with genome reorganization, neuropeptides, and energy mobilization. We discuss how these findings could be related to recent outbreaks and suggest how new efforts directed to better understand *P. guildinii* population dynamics.

## Introduction

The red-banded stink bug, *Piezodorus guildinii* (Westwood), or the ‘percevejo-verde-pequeno’ as it is known in Brazil, is one of the most important pests of soybean crops [*Glycinia max* (L.) Merr.], and along with *Nezara viridula* (L.) and *Euschistus heros* (F.) is responsible for significant damage in most cultivated areas of the New World^1,2^. *Piezodorus guildinii* was first discovered on the Caribbean island of St. Vincent, and its geographical distribution currently extends from Uruguay and northern Argentina to the southeastern region of the United States along the Gulf of Mexico, as well as Arkansas and South Carolina^1,3–5^. In contrast to Argentina and Uruguay where this insect is listed as the most important pest of soybean^5^, it is only sporadically reported as a problem in Brazil, and historically had limited impact in North America^6–10^. In recent years, however, *P. guildinii* has expanded its range and is becoming a more significant economic pest of crops in the U.S.^2^.

Specific aspects of *P. guildinii* population dynamics in North America are still not fully elucidated, but well-established populations and breeding sites in Florida and Mexico have been reported since the 1960s^6,8,9^. In recent years, great yield losses caused by the stink bugs complex —in which *P. guildinii* was the most abundant species—have been reported in Mississippi and Arkansas soybean fields^11^. The reason behind this apparent range expansion and recurrent outbreaks are not clear, and more information regarding the movement dynamics will be vital to understanding the recent appearance in southern states^11,12^. Interestingly, *P. guildinii* was not reported before the year 2000 in Louisiana, but now it is one of the major concerns for soybean producers^2,4^. One of the main hypotheses to explain recent attacks in Arkansas and Missouri is the seasonal movement from Louisiana, and possibly the influence of mild winters building larger populations^3,4,13^. However, these hypotheses have not been tested, and more data are needed to provide supporting evidence. It is possible that movement and adaptations to cold temperature are primary drives and can explain *P. guildinii* success colonizing and displacing other stink bug species^14^. Another hypothesis is that global climate change might affect the migration pattern of this species^15^.

In South America, particularly in Brazil, the rise in importance of *P. guildinii* has been directly linked to the agriculture technological revolution during the 1970s and subsequent soybean area expansion to central and northern lands^8^. Drastic changes in natural ecosystems and, to a large extent, substitution for extensively cultivated fields, correlates with first reports of *N. viridula* and *P. guildinii* in soybean^2^. During the following years, *P. guildinii* displaced *N. viridula* and became the primary pest in some areas of Brazil, but the reasons underlying this ecological dominance shifts are also not clear. Nowadays *E. heros* is undoubtedly the most abundant stinkbug species in soybean crops in Brazil, but *P. guildinii* damage is more severe to the soybean buds causing greater economic losses when they reach high population levels. Understanding more about *P. guildinii* populations in South America may provide insight on how to improve and develop new long-term sustainable pest control tactics. It can also improve our understanding of what could be causing range expansion and the dominance shifts within the stink bug complex in North and South America.

**Figure 1:**
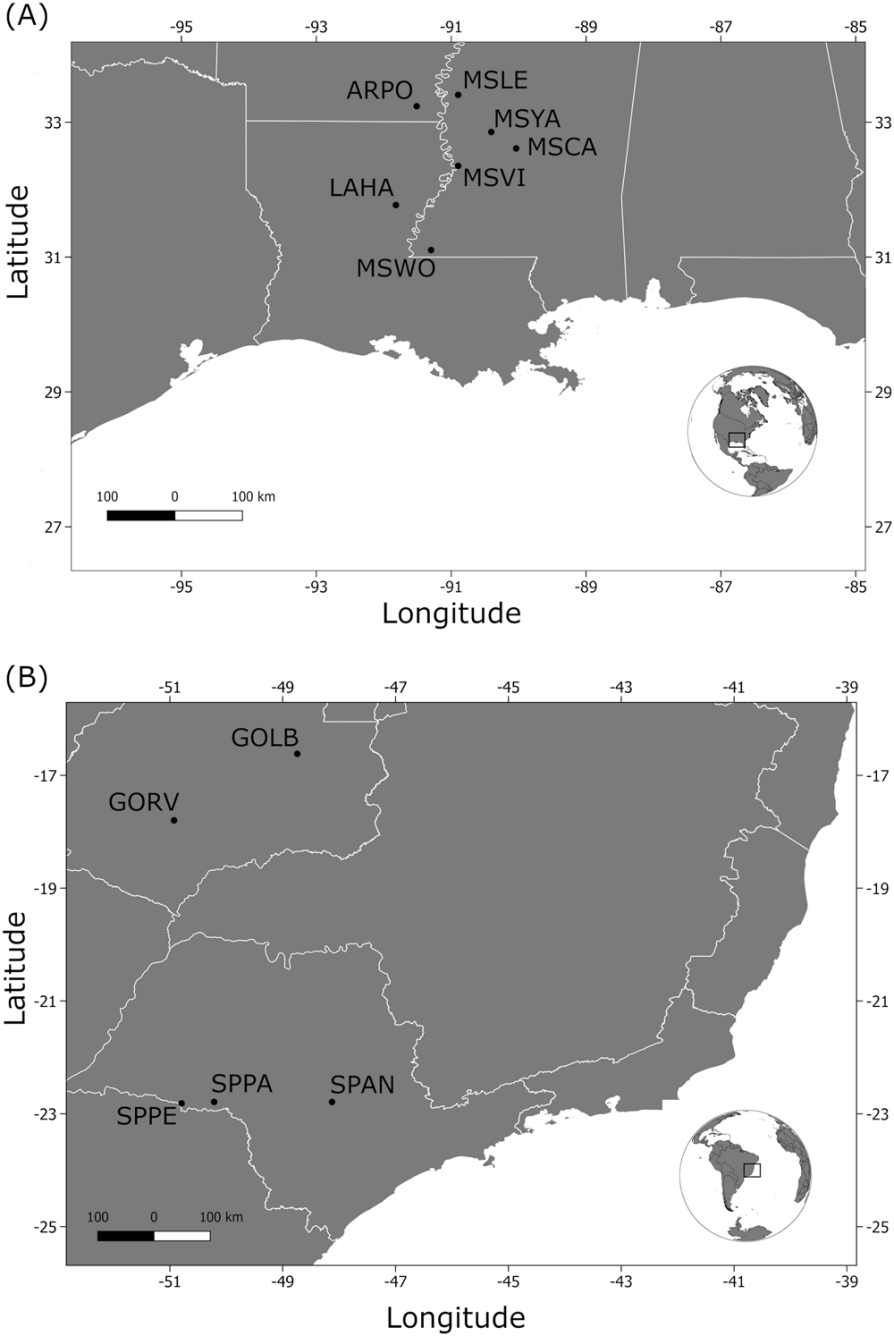
Map of *P. guildinii* sampling locations. On the top panel, North American locations including Louisiana (LAHA), Mississippi (MSWO, MSVI, MSCA, MSYA, and MSLE), and Arkansas (ARPO) from four different crop seasons, and South American locations (bottom panel) including Goiás (GOVR, GOLB) and Sao Paulo (SPAN, SPPA, and SPPE) collected during the 2016 crop season. Shapefiles were obtained at http://thematicmapping.org/.

In this study, we used over 4,083 single nucleotide polymorphisms (SNPs), generated by the genotyping-by-sequencing (GBS) methodology, to study *P. guildinii* populations collecting in five locations in Brazil, and seven locations in the U.S (Fig. 1). Our primary goal was to study how populations are connected at a fine and broad scale, to improve the understanding of the dispersal patterns of *P. guildinii* populations. We were also interested in studying how natural selection might have operated in different environmental conditions such as those found in North and South America. To the best of our knowledge, this is the first population genetic study explicitly targeting this group of insects which is becoming increasingly important in many areas of the American continent.

## Results

### SNP discovery and data processing

A total of 556,039 loci were genotyped in 159 individuals from 12 locations across the U.S. during four different crop seasons (2012, 2013, 2015, and 2016) and in Brazil during the crop season of 2016. Effective coverage per-sample was 12.2x (SD = 11.4x, min = 3.0x, max = 59.2x) per locus and the mean number of sites per locus of 100 sites. After filtering loci according to its minimum required frequency in a population, minor allele frequency (MAF), and maximum observed heterozygosity (MOH), a total of 1,337 loci containing 4,083 variant sites were retained for downstream population analyses. For structure analysis, only one SNP per locus was retained to avoid linkage bias.

### Diversity indexes

When considering all nucleotide positions (i.e., variants and non-variants), estimates for U.S. and Brazilian population have shown similar values for observed heterozygosity (H_O_ = 0.002 ± 0.000) and expected heterozygosity (H_E_ = 0.003 ± 0.000), nucleotide diversity (π = 0.003 ± 0.00), and inbreeding coefficient (F_IS_(_US_) = 0.005 ± 0.013 vs. F_IS_(_BR_) = 0.002 ± 0.008). Comparing locations within Brazil, the genetic diversity values showed no significant variation across the geographic range (Supplementary Tables S2 and S3). Within the U.S., genetic diversity parameters also did not show any significant differences considering geographical locations, crop seasons, or host plant at 5% probability (Supplementary Tables S2 and S3).

### F_ST_ and population structure

**Figure 2:**
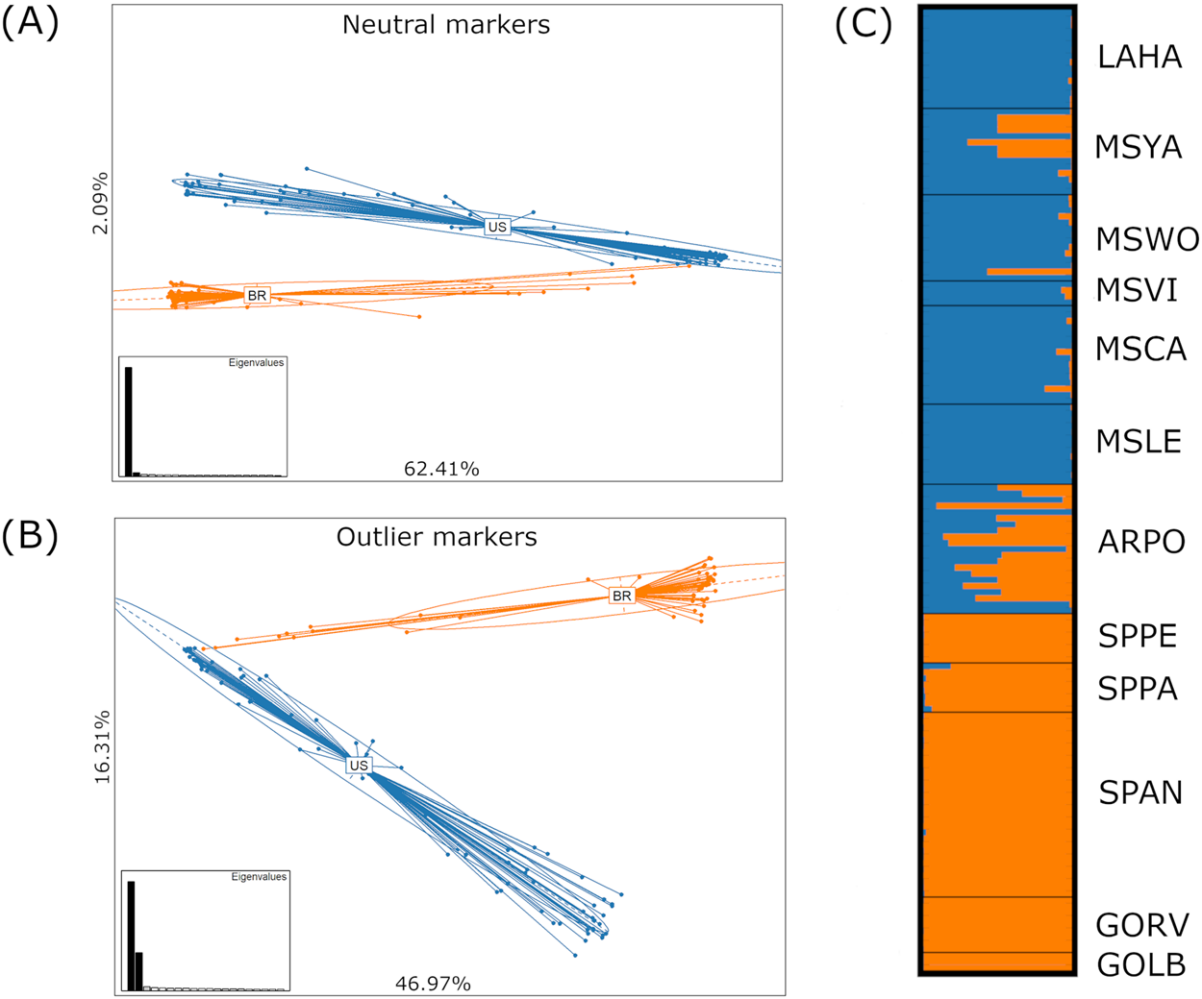
Principal component analysis (PCA) performed with 725 neutral markers (**A**) and 203 outlier markers detected by Bayescan (**B**). (**C**) Structure plot of *P. guildinii* of location in the United States and Brazil showing two levels of structure. Vertical bars represent individuals whose genotype have been portioned into 2 (K = 2) (between countries).

**Figure 3:**
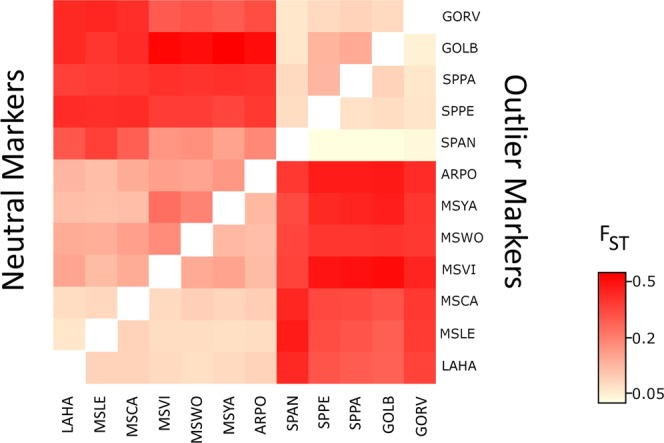
F_ST_ population dendrogram and heatmap based on F_ST_ values among the 12 *P. guilidinii* sampling locations using only neutral markers (**A**) and outlier SNPs. (**B**) Lighter colors represent less degree of differentiation in F_ST_ values, while darker red colors represent greater degree.

Based on the Evanno method, results from the STRUCTURE analyses using 725 neutral markers indicate that K = 2 was the higher level of hierarchical structure in which all Brazilian sampling locations were grouped in a single cluster. On the other hand, U.S. samples referent to 2012, 2013, 2015, and 2016 crop seasons were all grouped in a second cluster (Fig. 2C). The hypothesis that genetic differentiation would be more pronounced at regional level was confirmed by the AMOVA (F_CT_ = 0.26, p-value = 0.000), PCA, and F_ST_ results (Figs 2A and 3A, Supplementary Table S4). Interestingly, PCA and F_ST_ results based on SNP outliers revealed a similar pattern of cluster formation compared to neutral markers (Figs 2B and 3B), which suggests that genetic differentiation observed in natural populations is not only due stochastic processes (i.e., random drift) but also a product of adaptive evolution.

Calculations based on SNP outliers produced greater F_ST_ values compared to neutral markers for the same pairwise comparisons (Fig. 3, Supplementary Tables S5 and S6). This greater degree of differentiation produced when F_ST_ was calculated with outliers can be illustrated for the pairwise comparison between GOLB and MSVI; F_ST_ based on SNP outliers produced an estimation of 0.59 compared to 0.27 when only neutrals markers were used (Supplementary Table S6). To investigate the presence of levels of substructure considering that our sample contains insects from different crop seasons and hosts, we evaluated genetic partition between groups in higher values of K. All Brazilian locations behaved as a single homogeneous cluster even for higher values of K, while U.S. populations showed signs of a more heterogeneous and more admixture composition (Supplementary Fig. S6).

**Figure 4:**
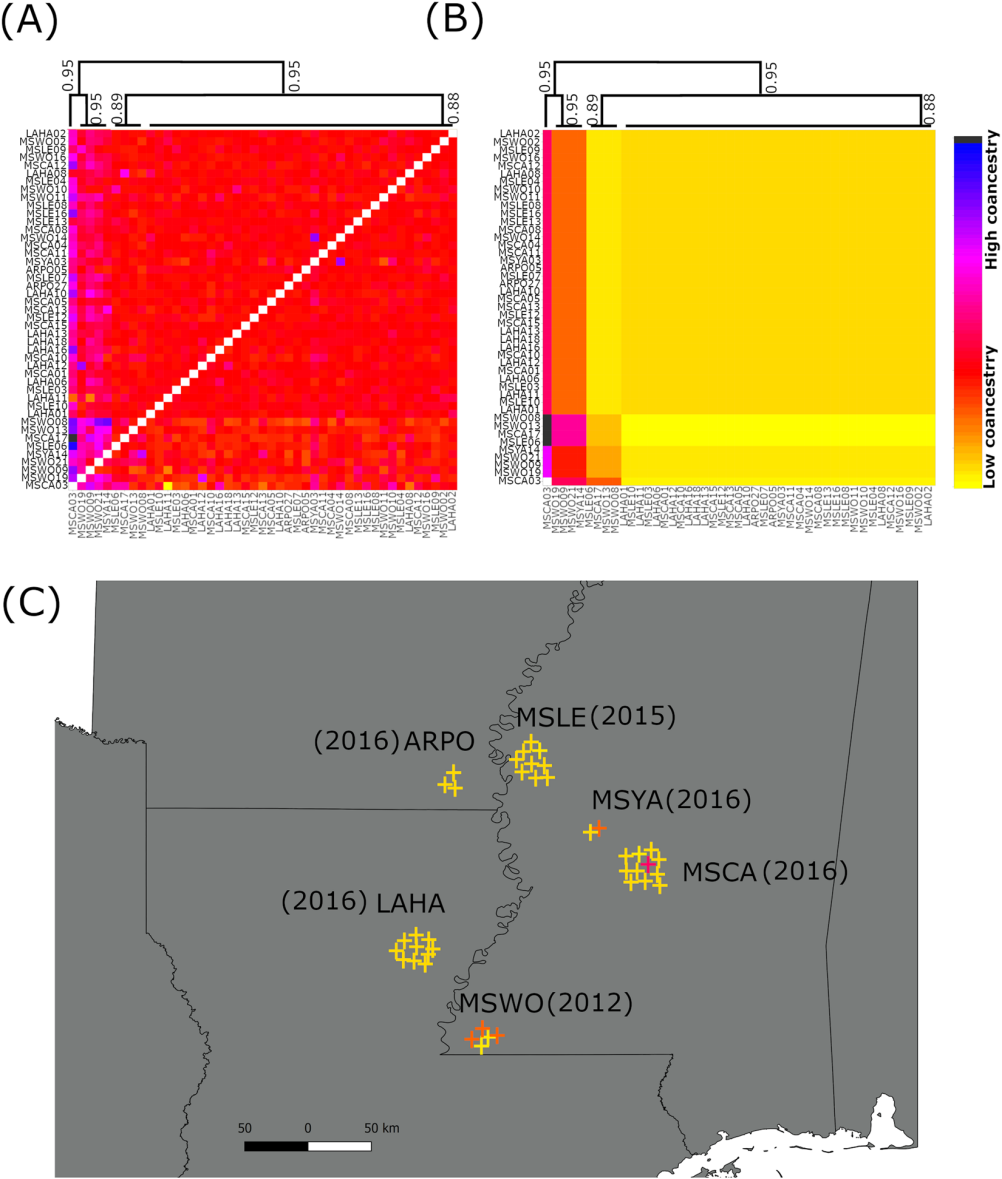
U.S. population structure according to fineRADstructure. (**A**) Coancestry matrix and population average matrix (**B**) were constructed based on six sample locations from three different crop seasons (2012, 2015, 2016). The collection performed in 2015 was remove from the analysis due to high levels of missing data. The different colors indicate different degrees of coancestry and the trees show putative relation between individuals based on shared coancestry. (**C**) Structure at fine scale based on fineRADstructure results. Crosses represent a single sample colored according to the prevalent coancestry relation with other samples found in (**B**).

Analysis with fineSTRUCTURE confirmed the presence of multiple groups of individuals sharing similar coancestry (i.e., gene correlation among individuals within-group) mixed at varies part of the sampling range in the U.S. The results indicate that the samples were collected in an area with an influx of individuals from different genetic backgrounds (i.e., high coancestry might indicate different origins of donors). Most samples collected in Mississippi were likely to result in multiple sources infestation (Fig. 4). The most admixture sample was MSWO collected in 2012; however, the least heterogeneous samples were also found in the state of Missouri, MSCA and MSYA (2016), (Fig. 4C).

### Outlier detection and candidate annotation

**Figure 5:**
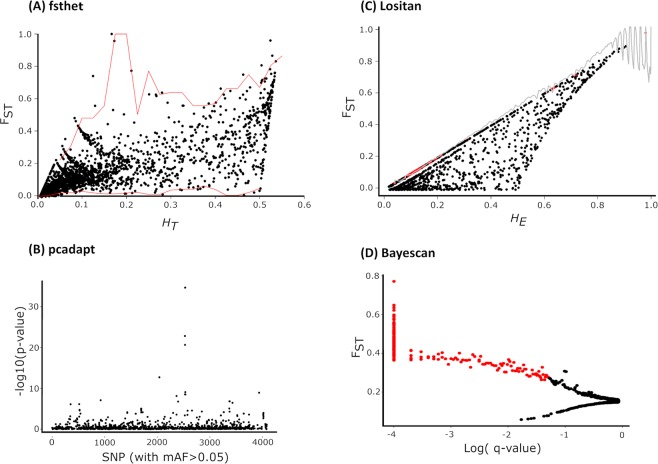
Detection of outlier SNP under positive selection using multiple algorithms are contrasting U.S. and Brazilian populations. (**A**) *fsthet* using a threshold of 0.05, (**B**) *pcadapt* using a threshold of 0.05, (**C**) Lositan using a confidence interval of 0.99 and a false discovery rate of (FDR) 0.01, and (**D**) Bayescan using FDR of 1%.

**Figure 6:**
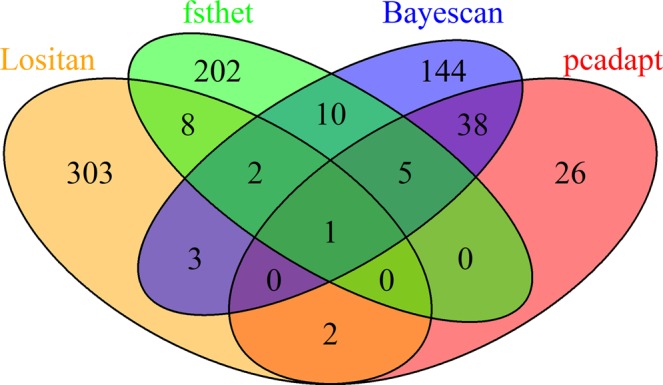
Venn diagram showing the number of unique and shared SNPs putatively under positive selection based on the comparison between U.S. and Brazilian populations.

All four methods used detected outliers SNPs putatively under positive selection when the U.S. and Brazil populations were contrasted (Fig. 5). The greatest number of candidates was detected by Lositan (319, ~8%) followed by *fsthet* (228, ~5.6%), Bayescan (203, ~5%), and *pcadapt* (72, 1.8%). Additionally, Bayescan detected 2,470 markers under balancing selection. Considering all methods, a total of 485 (~12%) outlier SNPs, out of which all programs concurred on a single marker (0.02%) (Fig. 6). The greatest concordance between the two methods was between Bayescan and *pcadapt* (44 outliers) while Lositan (303 outliers) was had a high rate of uniquely flagged SNPs (Fig. 5).

BLASTX analysis based on 485 candidate GBS loci containing outliers has successfully identified 123 loci under positive selection (10.5%) corresponding to known proteins in the NCBI database (e-value < 1.0 × E^−3^). A total of 58 were successfully mapped and 30 annotated. The only outlier identified concomitantly by the four methods was not successfully mapped. A great number of sequence descriptions were related to the genome reorganization such as retrotransposable elements, transposase, translocon, mobile elements, and reverse transcriptases. A small number of sequence descriptions was related to neuropeptides, and energy mobilization such as allatostatin-A receptor-like, neuropeptide CCHamide-1 receptor, and octopamine receptor.

Considering only Blastx results from *pcadapt* and Bayescan, the most conservative methods, a putative serine/threonine-protein kinase, and lipase 1-like isoform were present in both lists of results. Besides, Blast results from Bayescan also identified a carboxylesterase-6-like protein (e-value < 3.09 × E^−4^) as putative loci under selection.

## Discussion

In the present study, we have used genotyping-by-sequencing to examine the relation between and within populations of *P. guildinii* in North and South America^16^. By sequencing hundreds of markers, we were able to make inferences about population divergence, genetic structure, and the nature of the adaptative process shaping *P. guildinii* populations in the U.S and Brazil. Considering exclusively neutral markers, we gathered evidence that U.S. and Brazilian populations constitute two well differentiated gene pools that have remained allopatric separated with limited admixture throughout the years. Given the geographic distance, the presence of physical barriers, and differences in environmental conditions, the existence of well-differentiated genetic clusters was within our previous expectations. However, that is not always the case when it comes to insect pests that often reach global distribution with a history of recurrent invasions as is the case for the *Nezara viridula* and *Halyomorpha halys*^17–19^. A few locations, however, have showed signs of gene pool admixture (e.g. APRO and MSYA), which could suggest that the two gene pools are not completely isolated. It is nonetheless a fundamental step to study pest insect populations gather more information about their evolutionary history and to monitor the changes over the time, in particular in scenarios where range expansion and risk of invasion are present^20,21^.

Until now, the range of the geographic distribution of *P. guildinii* has been restricted to America, even though, the *Piezodorus* genus can be found distributed worldwide^1,22^. Notably, other species of the genus are also considered important pests of cultivated crops, but the evolutionary aspects of other species of the group are also poorly understood^1^. Even though at this point we have no information about morphological similarities or the degree of mating compatibility between the two populations, the differentiation between the two populations should be considered in further studies involving the Piezodorini group in Neotropical and Nearctic regions. Looking more closely at the chronology of reports of *P. guildinii* occurrence, data suggest that the increase in abundance is linked to human occupation of the continent and more recently to the transformation of the environment related to soybean crops. *Piezodorus guildinii* was initially described using material collected from the St. Vincent island in 1837 in the Caribbean Sea but rarelly reported in Brazil until the early 1970s^2^. In the U.S., the first reports about *P. guildinii* dated the early 1960s, but just recently this species became a significant problem^6–8^. We do not have details of the location and time where the *P. guildinii* diverged from other species of the group, but the chronology of reported dates suggests that the Central American and the Caribbean region might be the center of origin/differentiation of this group. If that is the case, then we can expect highly diverse populations in that region. However, this hypothesis still needs to be supported by data and more research is needed to access the levels of genetic diversity of insects from that region.

Resident populations of *P. guildinii* have been outlined in Florida and Texas for a much longer time than in Louisiana, Mississippi, and Arkansas, which may indicate the presence of more isolated gene pools in other areas of the U.S.^11,12,23^ Our analysis of structure in finer scales considering four different crop seasons give support to the idea that the Coastal plain regions are indeed a region with high levels of genetic admixture, and we can, therefore, hypothesize that more ancestral populations could be found at the extremes of the range, but our data does not allow us to draw any conclusion in this aspect. The heterogeneity seems to be more associated with the geographical location than the time of the collection or to the crop type. That fact that locations more to the east and the south of Mississippi had a more mixed genetic composition might be additional support for a possible greater genetic differentiation when a broader scale in the U.S. is considered. A broader sampling scheme that includes Mexico, Texas, Georgia, and Florida would be necessary to address the specific question regarding the source of migrants and the spatial structure within the U.S.

We have not found significant differences in the population-level genetic diversity values. The overall nucleotide diversity estimated harbored moderate levels of polymorphism (~3%) across the genome regardless of the sampling location and was comparable in both countries. The analysis of genetic partition within clusters (i.e., K = 3) and coancestry analysis suggested differences in the local dynamics even when we considered only insects collected in 2016. It is likely that demographic structure to be stable in Brazil with persistent populations over the time but unstable in the U.S. compromise of a great number of populations that last for only a few generations in certain cold areas^24–26^. One hypothesis to explain the presence of *P. guildinii* in higher latitudes (i.e., Arkansas) is the seasonal movement in years with mild winters from breeding sites in lower latitudes (i.e., southern Louisiana)^2^. Apart from previous expectation, the intraspecific distribution of the genetic diversity did not have a clear relationship with the latitude (i.e., no variation of diversity across the latitudinal range). A well-differentiated cluster and more homogenous sampling composition such as the ones observed for the MSLE and LAHA might be explained by a founding event or a single source of the infestation. *Piezodorus guildinii* are unlikely to establish a permanent population in those geographic locations due to the cold weather^25^. Higher values of genetic admixture and the presence of multiple coancestry groups might indicate movement from different directions instead of from a single source in the southern region.

In Brazil, in places where the insect can be found, its distribution is often associated with the presence of soybean fields^2^. No apparent pattern of spatial structure could be drawn from our data, which could initially be attributed to a high dispersal rate, the absence of physical barriers or simply the common ancestry of the sampled insects. Many important soybean regions (e.g., southern and southwest regions) are not represented in our sampled effort because 2016 was a year with very few reported incidences of *P. guildinii* in soybean fields in Brazil. Samples from Paraná, Mato Grosso, and Bahia must be included in future efforts as those represent key areas of soybean production that could provide a broader picture of the distribution of diversity across the geographic range in Brazil.

One of the questions we were interested here was to ask of the divergence between the two populations was primarily driven by stochastic processes such as genetic drift or due to adaptive evolution (i.e., natural selection). Our results clearly show that both neutral and adaptive evolution are components present in the history of this species. Gene flow, in certain cases, can be a constraint for local adaptation as the continuous influx of alleles from other populations drives the average population fitness down to suboptimum levels^27^. It is also likely the case that the absence of gene flow allowed the allopatric populations to evolve independently in many aspects, such as regarding physiological and behavioral attributes. Therefore, natural selection can both prevent or establish local differences more effectively than genetic drift^27^, which can, at least in part, explain why F_ST_ values calculated based on outlier SNPs were substantially higher compared to estimates based on neutral markers. However, it is challenging to pointing out which aspect of the environment—if climate, host distribution, or pesticide usage—is the most important driver of the adaptive response of *P. guildinii* populations. Understanding how pest populations are changing over time may help us explaining outbreaks or recent changes in ecological dynamics, including the ability to compete with other pest species^28^.

The annotation of outlier-containing sequences revealed several functional genes under directional and balancing selection based on four different methods. A simple distinction can be made about the two selection regimes; while in directional selection one phenotypic form in a population has a higher probability of advancing in following generations, in balancing selection more than one form can be favored (i.e., heterozygote advantage or frequency-dependent selection)^29^. In practical terms, the directional selection will cause populations to diverge (i.e., high F_ST_ between populations) whereas in balancing selection the distance between population might be shortened^30^. When we consider only loci putatively under directional selection, only one non-annotated marker was concurrently listed by all four methods used. The outlier detection methods vary in the ability to detect true positives, and the most appropriate method will depend on the degree of structure, true populations, and the degree of gene flow between populations^31^. Methods based on Bayesian approach (i.e., Bayescan) have shown least type I error, but methods that take in account genetic structure (i.e., *pcadapt*) can also be powerful tools to detect candidates^32–34^. In our data set, Bayescan and *pcadapt* were the methods with the greatest overlap suggesting good concordance between the two. Serine/threonine-protein kinase and lipase 1-like isoform X2 were the two successfully annotated loci candidates listed by the two methods, but functional studies are necessary to validate and to demonstrate the role of those candidates for the two populations.

Sequences were often involved in the genome reorganization, neuropeptides, and energy mobilization associated with lipid metabolism. Processes related to the genome reorganization can cause a further divergence between the groups even though the adaptive importance of those processes are still not clear to this moment^35^. We can pose the following questions: what are the ecological processes involved in genetic differentiation of the U.S. and Brazilian populations? Is the differentiation primarily driven by environmental conditions such as temperature and humidity or by resources such as host plants and pesticides? The annotation of putative loci suggests that the differentiation of the two populations involves a myriad of processes ranging from regulation of cellular processes, signaling, energy mobilization, and the regulation of primary metabolism. The annotation of the candidates provides evidence for the possible ecological importance of those processed in herbivory and movement. However, only a small portion of the outlier SNPs was successfully detected, compared to the NCBI database, and annotated, and therefore, we cannot disregard factors related to climate as a potential driver of the population differentiation.

In conclusion, current findings show that at least two well-structured populations occupy areas in North and South America. The differentiation was both caused by random drift and adaptive evolution. At the fine scale, high levels of population admixture were observed in the U.S., but not in Brazil suggesting a more heterogeneous and of mixed ancestry in the northern hemisphere. A broader sampling is needed to address the specific question of movement at a finer scale in both in Brazil and the U.S. Our data also suggests that natural selection is acting upon a variety of metabolic processes and it is likely related to genome reorganization and energy metabolism (i.e., lipids and carbohydrates) rather than climate adaptation. Further studies are necessary to conclude a causal association between what we have found here with broader ecological processes such as range expansion and ecological dominance shifts.

## Material and Methods

### Sampling

Red-banded stink bugs were sampled (Fig. 1, Table 1) from seven locations in the U.S. (a total of 128 insects), and five locations in Brazil (a total of 59 insects). Most samples were collected between May 2016 and September 2016, but we have also included samples from other planting seasons such as 2012 (MSWO), 2013 (MSVI), and 2015 (MSLE) from sites that experienced red-banded infestations during past crop seasons in the U.S. Adult insects were collected from soybean fields using sweep nets and were positively identified by the examination of the abdomen. Sampled insects were placed in 1.5 ml polypropylene microcentrifuge tubes (Eppendorf) tubes with 98% ethanol and stored at −80 °C until further processing.

**Table 1:** Sampling location information, coordinates, and the number of individuals successfully genotyped (N_GEN_) of *P. guildinii* by country.

LOC	Code	Date	Host	Latitude	Longitude	N_GEN_
Harrisonburg, LA	LAHA	4/19/2016	Crimson/Clover	31°46′19″N	91°49′17″W	20
Leland, MS	MSLE	10/15/2015	Soybean	33°24′51″N	90°53′51″W	16
Canton, MS	MSCA	4/27/2016	Crimson/Clover	32°36′45″N	90°02′12″W	17
Vicksburg, MS	MSVI	7/29/2013	Soybean	32°21′09″N	90°52′40″W	9
Woodville, MS	MSWO	6/21/2012	Soybean	31°06′16″N	90°17′58″W	21
Yazzo, MS	MSYA	4/27/2016	Crimson/Clover	32°51′18″N	90°24′20″W	14
Portland, AR	ARPO	4/29/2016	Crimson/Clover	33°14′12″N	91°30′42″W	31
Anhembi, SP	SPAN	3/2016	Soybean	22°47′22″S	48°07′38″W	30
Pedrinha, SP	SPPE	3/2016	Soybean	22°48′54″S	50°47′38″W	9
Palmital, SP	SPPA	3/2016	Soybean	22°47′20″S	50°13′03″W	8
Leopoldo Bulhões, GO	GOLB	3/2016	Soybean	16°37′09″S	48°44′37″W	3
Rio Verde, GO	GORV	3/2016	Soybean	17°47′53″S	50°55′41″W	9

### DNA extraction, quality control, and RAD library preparation and sequencing

We extracted DNA from the head and legs from randomly selected individuals using cetyltrimethylammonium bromide (CTAB)-based protocol adjusting proteinase K concentrations^36^. DNA quality and approximate quantity were assayed by agarose electrophoresis gel (1% w/v) stained with SYBR Safe DNA Gel Stain (Invitrogen), visually compared with lambda DNA standards (Invitrogen), and used to construct GBS libraries, similar to those previously described by Poland *et al*.^16^. DNA concentrations were determined more precisely using picogreen (Molecular Probes, Eugene, Oregon) and a Synergy HT (BioTek, Winooski, Vermont) microplate readers, and adjusted to approximately 50 ng/μl. Five microliters (~250 ng) were pipetted into a new set of 96-well plates containing 2.5 μl 0.1 μM specific DNA barcoded (5–10 bp) *Pst*I adaptors. The restriction enzymes *Pst*I (New England Biolabs, Whitby, ON, Canada) and *Msp*1 (New England Biolabs) were used to digest the DNA and reduce genome complexity. The barcoded *Pst*I adapters (CTGCAG) and a common non-barcoded *Msp*I adapter (CCGG) were ligated to the digested DNA and amplified by PCR to create a sequencing library following Poland *et al*.^16^. The resulting library was single-end sequenced to 150 nucleotides on a single lane using the Illumina HiSeq. 4000 sequencing kit v1 (Illumina, Inc., San Diego, CA, USA), and the fastq files were demultiplexed with the bcl2fastq v2.17.1.14 (Illumina) by the Roy J. Carver Biotechnology Center at the University of Illinois at Urbana-Champaign producing over 133 million raw reads.

### SNP discovery and genotyping

We processed the Illumina raw single-end reads using the program *process-radtags* in STACKS v.2.4^37^. The original Illumina data consisted of 133,097,998 reads pooled in a single file. *Process-radtags* uses information from the individual barcodes, and the RAD cut site to demultiplex sequence data assigning reads to the correct sample ID. Reads with uncalled bases and low-quality sequence reads were removed. RAD-tags and barcodes were rescued during demultiplexing, allowing a single mismatch while rescuing single-end barcodes. A total of 284,641 reads were discarded as they were low quality or contained ambiguous barcode or RAD-Tags. Sequence reads were truncated to 100 nucleotides long.

After demultiplexing, we used the *de novo* approach to assembly loci and call SNPs. The number of mismatches allowed between stacks while merging them into putative loci (*ustacks*, *M*) and during the construction of stacks catalog (*cstacks*, *n*) have shown to be critical steps in *de novo* analyses and are dependent on the natural levels of polymorphism found in the sample (i.e., biological information) and the technical approach used to produce libraries^38^. We investigated the impact of *M* and *n* is the number of retained loci and SNPs while varying the percentage of individuals required to possess a certain locus (*i.e*., from *r10* to *r60*) in STACKS population module. First, we assumed *M* = *n* and considered only loci with a minimum depth of coverage of 3 × (*m* = 3) to build putative RAD-loci^38,39^. Loci and SNP distribution plateaus between *Mn*3 and *Mn*4, therefore *M* and *n* were set to 4. To minimize the impact of missing while still trying to extract reliable biological information from available samples, we investigated retained SNP and loci distribution for different values of percentage of individuals required to have a locus for it to be processed in population module (*r*). Our criteria to select *r* was based on the maximum possible values until it reaches an approximate value of ~1,500 loci, which in this case was *r55*.

Both common and rare alleles can help us detecting important demographic and ecological phenomena in natural systems and therefore, be treated with great care. While common alleles can be used to detect bottlenecks, purifying selection, and population structure, high frequency of rare alleles may indicate population expansion, abnormal mutation rates, and gene flow in fine-scale^40^. However, steps during library preparation such as PCR, library sequencing, and bioinformatic treatment to the data can generate errors and create biases for the population genetics estimations^40^. To prevent biases created by the excess of singletons, we investigated the impact of the parameter that controls minor allele frequency (MAF) to the number of retained SNPs. No clear sign of an excess of singleton was detected in our dataset, but to ensure that no ‘artificial alleles’ would interfere in our estimates, we applied a 1% MAF filter (MAF < 0.01)^41^. Moreover, to minimize the influence of paralogs from the final dataset, we have limited the maximum observed heterozygosity (MOH) to 45%, after investigating the distribution of the parameter in our samples.

All descriptive diversity statistics (i.e., Expected heterozygosity (H_E_), nucleotide diversity (π), and F_IS_) and pairwise F_ST_ values were calculated using the population module of STACKS. A heatmap was produced to illustrate F_ST_ relations using the R package *RColorBrewer*. Results were saved in genepop, variant call format (VCF) and structure files and converted to other formats using PGDSPIDER 2.0^42^.

### Population clustering and differentiation

A model-based Bayesian method was implemented to inferred population structure using the program STRUCTURE v.2.3.4^43,44^. The input data set was treated to ensure that a single SNP per locus would be used thus minimizing biases from strong linkage disequilibrium between markers. Furthermore, input data was only composed by neutral markers determined in Bayescan (i.e., excluding markers under balancing and diversifying selection) and all significant outliers for diversifying selection flagged by Lositan, *fsthe*, and *pcadapt* removed (described in the next section). To run STRUCTURE, we assumed admixture and allele frequency correlation models, and the program was set to run 1.5 × 10^5^ burn-in followed by another 5 × 10^6^ Markov Chain Monte Carlo (MCMC) interactions. We simulated the number of clusters ranging from 1 to 15 (K = 1 to K = 15) with 15 repetitions for each value. The optimal K value was selected using the Evanno method implemented in Structure Harvest^45^. Multiple runs were averaged using CLUMPP 1.1.2 software^46^ and plotted using DISTRUCT^47^. To offer an additional support for the number of genetic clusters, we also used a non-model method based on principal component analysis (PCA) using *Adegenet* package in R. The mean of allele frequencies at a certain locus was used to when genotype data was missing with missing. Finally, the test the hypothesis of two genetic clusters (i.e., structure by country) we used an analysis of molecular variance (AMOVA) using all and exclusively neutral loci^48^. To estimate AMOVA’s F_ST_, we used pairwise differences distance matrix and 10,000 permutations to determine the significance of the parameter.

To investigate genetic structure in fine scale in the U.S., we used *fineRADstructure*^49–51^. The approach is based on nearest neighbor haplotype ‘coancestry’ in which a coancestry matrix is first created in RADpainter following the assignment individuals to putative populations using *fineSTRUCTURE*. Markov Chain Monte Carlo (MCMC) clustering algorithm ran for 1 × 10^6^ MCMC generations following an initial 1 × 10^6^ generation burn-in and figures were plotted using R script *fineRADstructurePlot.R* and *FinestructureLibrary.R* (https://github.com/millanek/fineRADstructure).

### Outlier detection and candidate annotation

We investigated loci putatively under selection using F_ST_-based tests to detect outlier loci candidates that could be associated with patterns of differentiation between populations given neutral expectations. The tests contrasted Brazil and U.S. populations, except for Bayescan that considered all locations separately. The first method was implemented in LOSITAN^52^ that is based on the relation between F_ST_ values and heterozygosity of each marker, comparing it to data simulation. Beaumont and Nichols (1996), and it is based on the infinite island model^53^. LOSITAN was set to run 1 × 10^6^ (100 × 1,000) simulations using a confidence interval of 0.99 and a false discovery rate of (FDR) 0.01. Neutral mean and force mean F_ST_ options were selected. However, LOSITAN has a comparatively high rate of false positives compared to Bayesian methods and can produce unusual pattern derived from F_ST_ and H_E_ mathematical relation^31,32^. Incline and skewed patterns often emerge when few independent genetic clusters with limited gene flow are present^31^. To mitigate this problem, we used an alternative method available in *fsthe* package in R that uses the raw empirical data to generate smoothed outlier plot. In addition to the two described methods, we use a PCA based method that takes into account population structure and can handle well high rates of missing data and admixture^34^. Lastly, we used a Bayesian approach in Bayescan using default parameters: 20 short pilot runs with 5,000 integration, burn-in set to 5 × 10^4^ and thinning interval and prior odds of 10. To select candidates, we used FDR of 1%^54^.

All RAD-loci containing the outlier SNP by the different methods were used as a query in nucleotide search BLASTx against the National Center for Biotechnology Information (NCBI) using Blast2Go suite following mapping and annotation steps using default configurations^55^.

### Ethical standard

Sampling was carried out following standard regulation and with the proper research permits issued by the Brazilian Federal Government Institution (Ministério do Meio Ambiente, Instituto Chico Mendes de Conservação da Biodiversidade – ICMBio/SISBIO: No. 44402 e 63459. The collection made during this study did not involve endangered species.

## Supplementary information

Supplementary information

## Data Availability

Supplementary material files such as structure, genepop, VCF files are available at FigShare (https://figshare.com/s/bd829a25dee73a235e14). We have also provided lists of outlier candidates with sequence ID and annotation for additional support of the information presented here. Any additional data can be provided upon request. Illumina generated reads of each individuals are available at the NCBI Sequence Read Archive (SRA) under accession number PRJNA559462. All data necessary to reproduce this study was provided as supplementary material or deposited at FigShare and NCBI-SRA. Any additional data or information are available from the corresponding author upon request.
