# Supplementary material for: Patterns of Genome-Wide Variation, Population Differentiation and SNP Discovery of the Red Banded Stink Bug (Piezodorus guildinii)
Source: Sci Rep. 2019 Oct 9;9:14480. doi: 10.1038/s41598-019-50999-z (PMC6785548; doi:10.1038/s41598-019-50999-z)
Supplement: Supplementary file 1 — Supplementary information [file 41598_2019_50999_MOESM1_ESM.docx]

**Supplementary material**

**Patterns of Genome-Wide Variation, Population Differentiation and SNP Discovery of the Red Banded Stink Bug (*Piezodorus guildinii*)**

Maria I. Zucchi, Erick M. G. Cordeiro, Clint Allen, Mariana Novello, João Paulo Gomes Viana, Patrick J. Brown, Shilpa Manjunatha, Celso Omoto, José Baldin Pinheiro, Steven J. Clough

Parameter selection for the de novo analysis in STACKS

**Table S1.** Integrating values for the distance allowed between two stacks (M) for data presented in at least 40% of the sampled (*r40*).

| Parameters | Assembled loci |
| --- | --- |
| M1m3n1 | 716,657 |
| M2m3n2 | 617,325 |
| M3m3n2 | 580,544 |
| M4m3n4 | 554,801 |
| M5m3n6 | 538,702 |
| M6m3n6 | 527,414 |
| M7m3n6 | 519,372 |


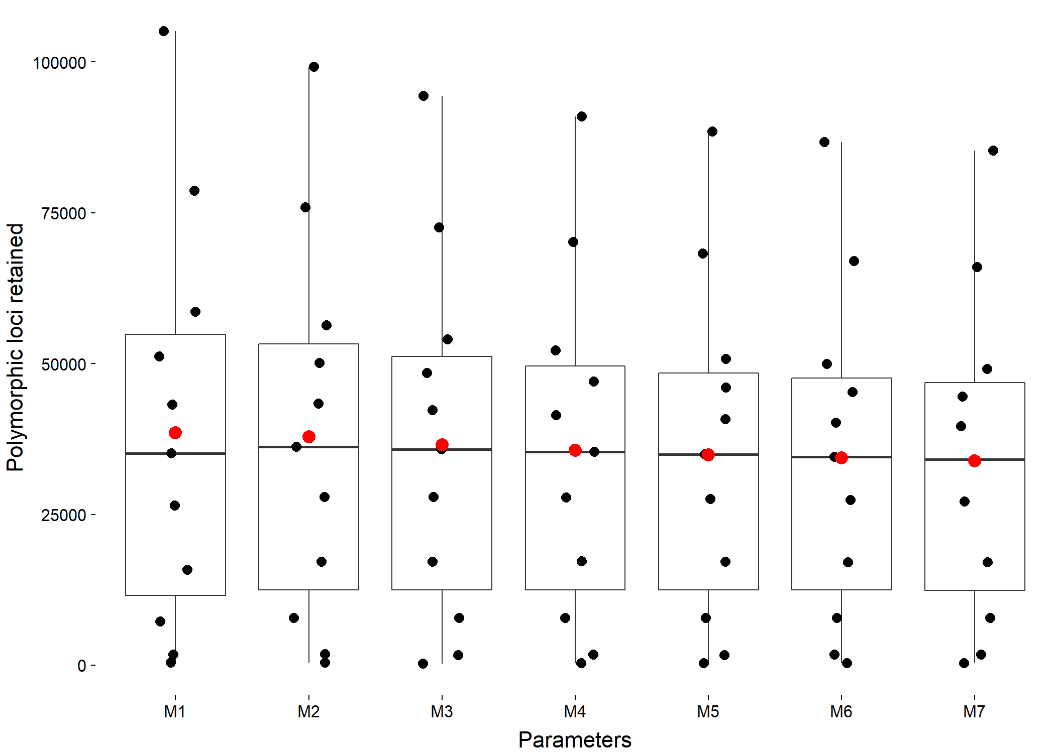


**Figure S1. Number of polymorphic loci retained in function of the distance allowed between two stacks (M).** The distribution of each value of M was generated by varying values for the minimum percentage of individuals in a population (-*r*) from 10% (*r10*) to 60% (*r60*). Red dot represents the mean. Mean values of M stabilize between M3 and M4.


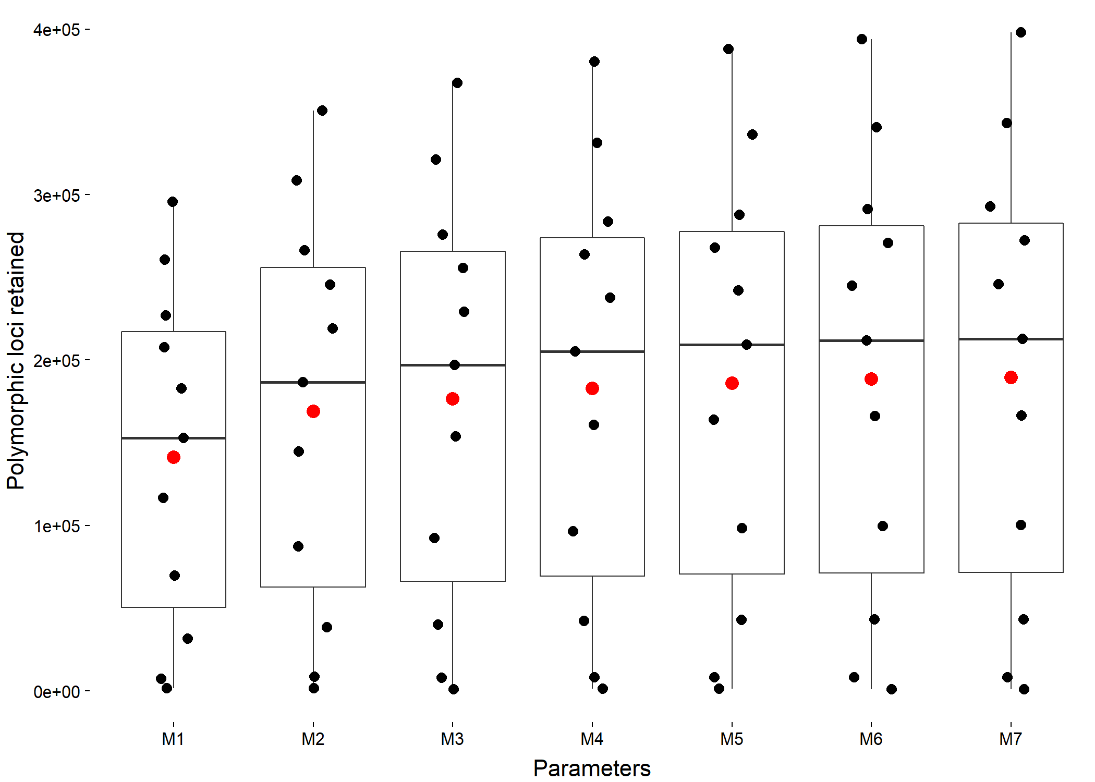


**Figure S2. Number of SNPs retained in function of the distance allowed between two stacks (M).** The distribution of each value of M was generated by varying values for the minimum percentage of individuals in a population (-*r*) from 10% (*r10*) to 60% (*r60*). Red dot represents the mean. Mean values of M plateau between M3 and M4.


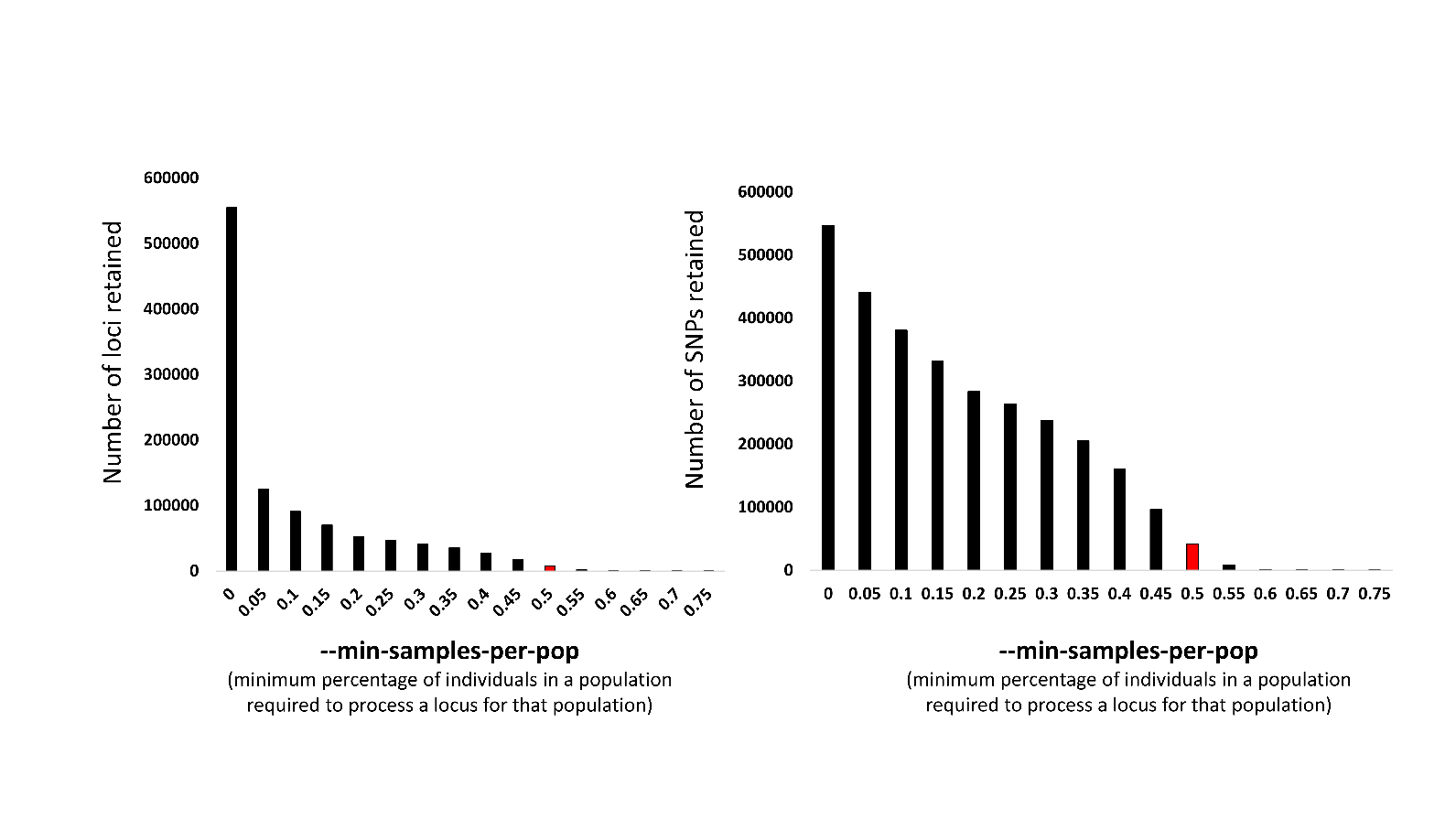


**Fig S3. Number of loci and SNPs retained in function of the minimum percentage of individuals in a population required to process a locus (*-r*).** Red bar represented the best tradeoff between missing rate and number of SNPs retained.


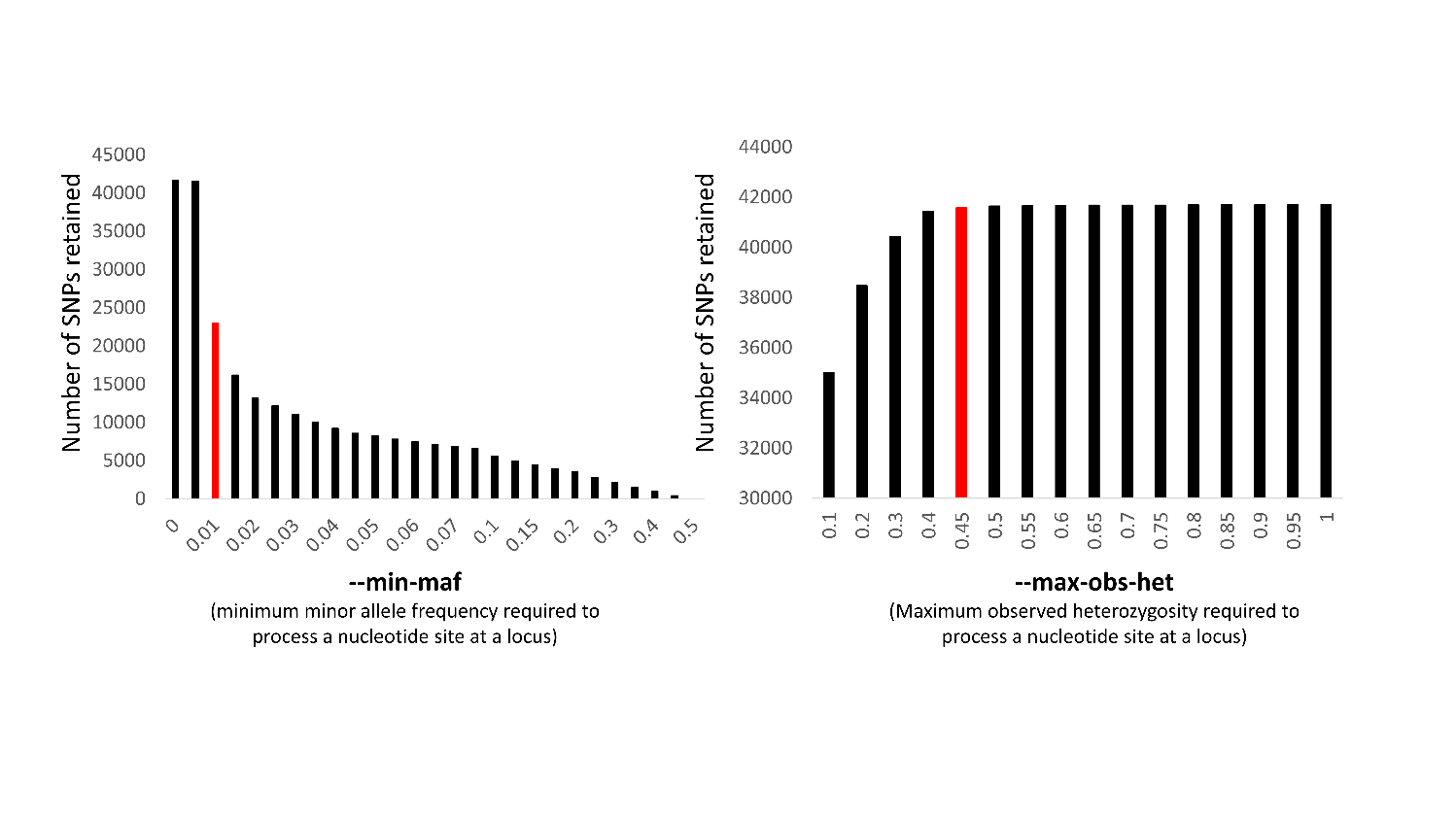


**Fig S4. A number of loci and SNPs retained in the function of the minimum minor allele frequency (*MAF*) and maximum observed heterozygosity (*MOH*).** The red bar for MAF represented the cut off of potential singletons and for the MOH represent when the parameter plateau.

**Tab. S2.** STACKS genetic diversity summary considering only variant positions.

| POP | H_O_ | H_E_ | π | F_IS_ |
| --- | --- | --- | --- | --- |
| LAHA (2016) | 0.065±0.002 | 0.086±0.002 | 0.091±0.002 | 0.079±0.025 |
| MSLE (2015) | 0.079±0.002 | 0.091±0.002 | 0.095±0.002 | 0.055±0.019 |
| MSCA (2016) | 0.064±0.002 | 0.083±0.002 | 0.089±0.002 | 0.076±0.024 |
| MSVI (2013) | 0.075±0.003 | 0.062±0.002 | 0.084±0.003 | 0.015±0.007 |
| MSWO (2012) | 0.040±0.002 | 0.063±0.002 | 0.074±0.003 | 0.075±0.030 |
| MSYA (2016) | 0.063±0.003 | 0.057±0.002 | 0.079±0.003 | 0.028±0.019 |
| ARPO (2016) | 0.082±0.003 | 0.076±0.002 | 0.092±0.003 | 0.019±0.014 |
| SPAN (2016) | 0.078±0.002 | 0.082±0.002 | 0.084±0.002 | 0.036±0.030 |
| SPPE (2016) | 0.066±0.002 | 0.071±0.002 | 0.080±0.002 | 0.035±0.020 |
| SPPA (2016) | 0.066±0.002 | 0.068±0.002 | 0.079±0.002 | 0.028±0.018 |
| GOLB (2016) | 0.073±0.003 | 0.066±0.002 | 0.080±0.003 | 0.012±0.004 |
| GORV (2016) | 0.074±0.002 | 0.077±0.002 | 0.082±0.002 | 0.026±0.013 |
| US | 0.067±0.002 | 0.090±0.002 | 0.092±0.002 | 0.149±0.066 |
| BR | 0.074±0.002 | 0.083±0.002 | 0.083±0.002 | 0.080±0.047 |

**Tab. S3.** STACKS genetic diversity summary considering only variant and non-variant positions.

| POP | H_O_ | H_E_ | π | F_IS_ |
| --- | --- | --- | --- | --- |
| LAHA (2016) | 0.002±0.000 | 0.003±0.000 | 0.003±0.000 | 0.002±0.004 |
| MSLE (2015) | 0.002±0.000 | 0.003±0.000 | 0.003±0.000 | 0.002±0.003 |
| MSCA (2016) | 0.002±0.000 | 0.003±0.000 | 0.003±0.000 | 0.002±0.004 |
| MSVI (2013) | 0.002±0.000 | 0.002±0.000 | 0.003±0.000 | 0.000±0.001 |
| MSWO (2012) | 0.001±0.000 | 0.002±0.000 | 0.002±0.000 | 0.002±0.005 |
| MSYA (2016) | 0.002±0.000 | 0.002±0.000 | 0.002±0.000 | 0.001±0.003 |
| ARPO (2016) | 0.002±0.000 | 0.002±0.000 | 0.003±0.000 | 0.001±0.002 |
| SPAN (2016) | 0.002±0.000 | 0.003±0.000 | 0.003±0.000 | 0.001±0.004 |
| SPPE (2016) | 0.002±0.000 | 0.002±0.000 | 0.002±0.000 | 0.001±0.003 |
| SPPA (2016) | 0.002±0.000 | 0.002±0.000 | 0.002±0.000 | 0.001±0.003 |
| GOLB (2016) | 0.002±0.000 | 0.002±0.000 | 0.002±0.000 | 0.000±0.001 |
| GORV (2016) | 0.002±0.000 | 0.002±0.000 | 0.002±0.000 | 0.001±0.002 |
| US | 0.002±0.000 | 0.003±0.000 | 0.003±0.000 | 0.005±0.013 |
| BR | 0.002±0.000 | 0.003±0.000 | 0.003±0.000 | 0.002±0.008 |

**Fig. 5.** Structure plot for K=2-6 based on 725 neutral makers. A single SNP per loci was used for the STRUCTURE analysis.


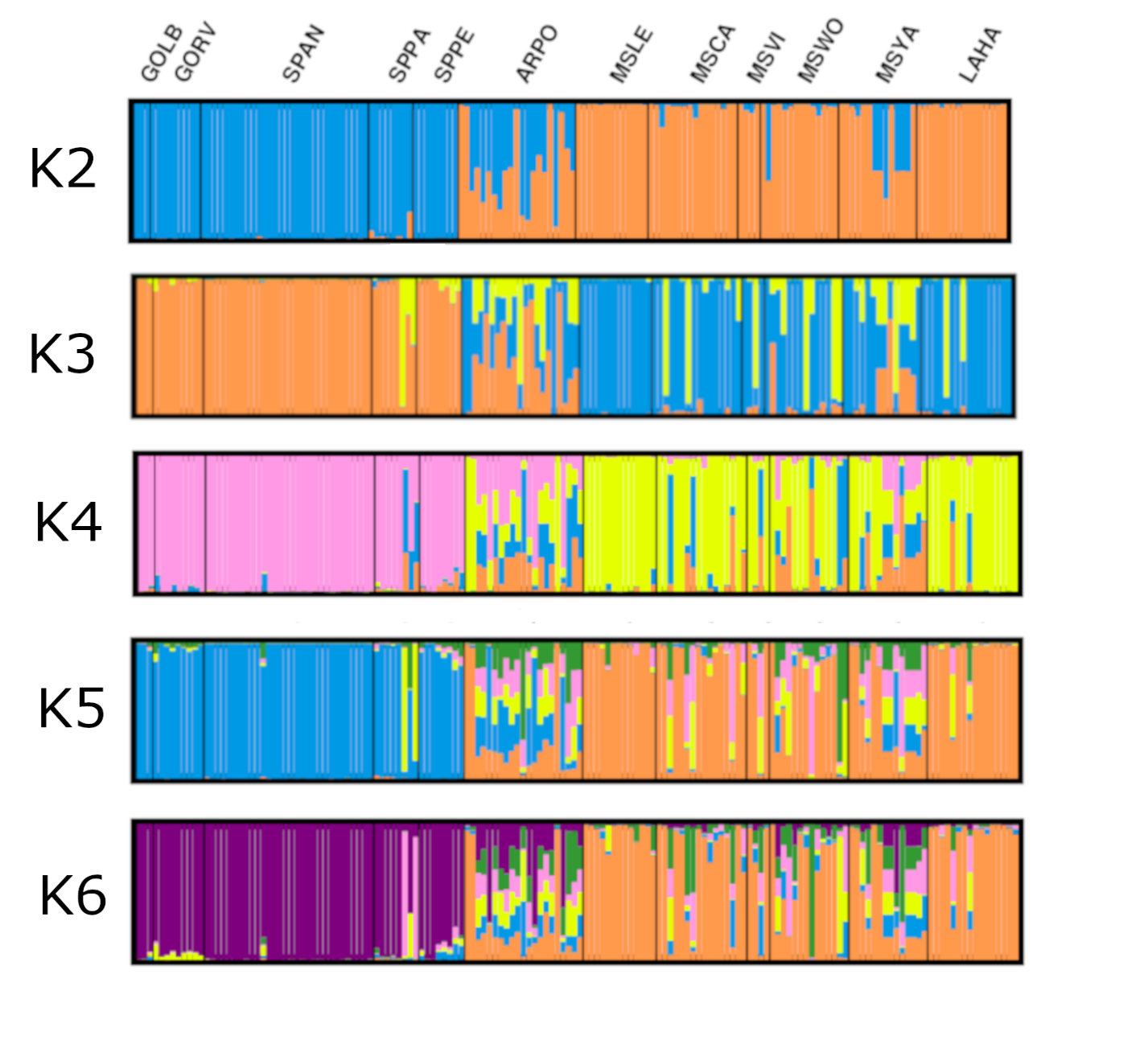


**Tab S4.** AMOVA table of *Piezodorus guildinni* populations based on 725 neutral markers

| **Source of variation** | **Sum of squares** | **Variance components** | **Percentage variation** |
| --- | --- | --- | --- |
| Among groups **(US vs. BR)** | 524.867 | 5.61 | 25.8 |
| Among populations within groups | 238.303 | 0.68 | 3.11 |
| Within populations | 2557.417 | 15.46 | 71.08 |
| Total | 3320.59 | 21.75 |  |

**Average- F-statistics over all loci**

F_ST_: 0.29 (p-value= 0.000)

F_SC_: 0.04 (p-value= 0.000)

F_CT_: 0.26 (p-value= 0.000)

**Tab S5.** Table showing pairwise F_ST_ values of *Piezodorus guildinni* populations based on 725 neutral markers

|  | LAHA | MSLE | MSCA | MSVI | MSWO | MSYA | ARPO | SPAN | SPPE | SPPA | GOLB | GORV |
| --- | --- | --- | --- | --- | --- | --- | --- | --- | --- | --- | --- | --- |
| LAHA(2016) |  | 0.042 | 0.047 | 0.073 | 0.07 | 0.061 | 0.065 | 0.109 | 0.129 | 0.12 | 0.131 | 0.131 |
| MSLE(2015) | 0.042 |  | 0.043 | 0.055 | 0.061 | 0.053 | 0.054 | 0.109 | 0.117 | 0.11 | 0.114 | 0.121 |
| MSCA(2016) | 0.047 | 0.043 |  | 0.073 | 0.079 | 0.065 | 0.074 | 0.11 | 0.135 | 0.129 | 0.135 | 0.133 |
| MSVI(2013) | 0.073 | 0.055 | 0.073 |  | 0.145 | 0.17 | 0.126 | 0.136 | 0.215 | 0.226 | 0.267 | 0.189 |
| MSWO(2012) | 0.07 | 0.061 | 0.079 | 0.145 |  | 0.13 | 0.11 | 0.123 | 0.173 | 0.179 | 0.203 | 0.16 |
| MSYA(2016) | 0.061 | 0.053 | 0.065 | 0.17 | 0.13 |  | 0.126 | 0.115 | 0.2 | 0.218 | 0.264 | 0.178 |
| ARPO(2016) | 0.065 | 0.054 | 0.074 | 0.126 | 0.11 | 0.126 |  | 0.125 | 0.178 | 0.18 | 0.206 | 0.163 |
| SPAN(2016) | 0.109 | 0.109 | 0.11 | 0.136 | 0.123 | 0.115 | 0.125 |  | 0.039 | 0.041 | 0.033 | 0.034 |
| SPPE(2016) | 0.129 | 0.117 | 0.135 | 0.215 | 0.173 | 0.2 | 0.178 | 0.039 |  | 0.093 | 0.094 | 0.067 |
| SPPA(2016) | 0.12 | 0.11 | 0.129 | 0.226 | 0.179 | 0.218 | 0.18 | 0.041 | 0.093 |  | 0.103 | 0.072 |
| GOLB(2016) | 0.131 | 0.114 | 0.135 | 0.267 | 0.203 | 0.264 | 0.206 | 0.033 | 0.094 | 0.103 |  | 0.056 |
| GORV(2016) | 0.131 | 0.121 | 0.133 | 0.189 | 0.16 | 0.178 | 0.163 | 0.034 | 0.067 | 0.072 | 0.056 |  |

**Tab. S6.** Table showing pairwise F_ST_ values of *Piezodorus guildinni* populations based on 203 outlier SNP markers.

|  | LAHA | MSLE | MSCA | MSVI | MSWO | MSYA | ARPO | SPAN | SPPE | SPPA | GOLB | GORV |
| --- | --- | --- | --- | --- | --- | --- | --- | --- | --- | --- | --- | --- |
| LAHA(2016) |  | 0.034 | 0.051 | 0.06 | 0.057 | 0.074 | 0.072 | 0.44 | 0.423 | 0.399 | 0.392 | 0.453 |
| MSLE(2015) | 0.034 |  | 0.052 | 0.05 | 0.061 | 0.062 | 0.05 | 0.465 | 0.438 | 0.415 | 0.398 | 0.468 |
| MSCA(2016) | 0.051 | 0.052 |  | 0.059 | 0.09 | 0.084 | 0.083 | 0.446 | 0.453 | 0.438 | 0.425 | 0.477 |
| MSVI(2013) | 0.06 | 0.05 | 0.059 |  | 0.166 | 0.196 | 0.126 | 0.384 | 0.57 | 0.579 | 0.599 | 0.523 |
| MSWO(2012) | 0.057 | 0.061 | 0.09 | 0.166 |  | 0.142 | 0.119 | 0.379 | 0.491 | 0.486 | 0.5 | 0.474 |
| MSYA(2016) | 0.074 | 0.062 | 0.084 | 0.196 | 0.142 |  | 0.136 | 0.364 | 0.521 | 0.531 | 0.549 | 0.48 |
| ARPO(2016) | 0.072 | 0.05 | 0.083 | 0.126 | 0.119 | 0.136 |  | 0.406 | 0.556 | 0.547 | 0.559 | 0.507 |
| SPAN(2016) | 0.44 | 0.465 | 0.446 | 0.384 | 0.379 | 0.364 | 0.406 |  | 0.026 | 0.023 | 0.013 | 0.017 |
| SPPE(2016) | 0.423 | 0.438 | 0.453 | 0.57 | 0.491 | 0.521 | 0.556 | 0.026 |  | 0.089 | 0.095 | 0.066 |
| SPPA(2016) | 0.399 | 0.415 | 0.438 | 0.579 | 0.486 | 0.531 | 0.547 | 0.023 | 0.089 |  | 0.117 | 0.061 |
| GOLB(2016) | 0.392 | 0.398 | 0.425 | 0.599 | 0.5 | 0.549 | 0.559 | 0.013 | 0.095 | 0.117 |  | 0.039 |
| GORV(2016) | 0.453 | 0.468 | 0.477 | 0.523 | 0.474 | 0.48 | 0.507 | 0.017 | 0.066 | 0.061 | 0.039 |  |
